# Left Ventricular Outflow Tract Obstruction From Proximal Intimo-Intimal Intussusception: A Fatal Complication of Acute Type A Aortic Dissection

**DOI:** 10.7759/cureus.116796

**Published:** 2026-09-23

**Authors:** Erich Burton, John C McGee, Neel Vahil, Trenton Wray

**Affiliations:** 1 Department of Emergency Medicine, Center for Adult Critical Care, University of New Mexico, Albuquerque, USA; 2 Department of Internal Medicine, Division of Pulmonary and Critical Care Medicine, University of New Mexico, Albuquerque, USA; 3 Department of Internal Medicine and Pulmonary and Critical Care, Johns Hopkins University, Baltimore, USA

**Keywords:** cardiac arrest, cardiac resuscitation, ehlers-danlos syndrome, transesophageal echocardiography (tee), type a aortic dissection

## Abstract

Acute type A aortic dissection is a highly time-sensitive cardiovascular emergency in which mechanical obstruction of the left ventricular outflow tract (LVOT) by a prolapsing intimo-intimal intussusception flap represents a rare and often fatal complication, producing severe hemodynamic compromise that may be refractory to conventional resuscitative measures. We report a 26-year-old woman with Ehlers-Danlos syndrome who was transferred for evaluation of a large proximal type A aortic dissection and suffered a witnessed cardiac arrest shortly after arrival. Transesophageal echocardiography (TEE) demonstrated a dissection flap prolapsing into the subvalvular LVOT during diastole, disrupting aortic valve coaptation and causing aortic insufficiency (AI), while moving anterograde during systole to obstruct forward flow. Despite transient return of spontaneous circulation, resuscitation ultimately failed, and veno-arterial extracorporeal membrane oxygenation was not pursued because of the underlying aortic dissection, AI, prolonged resuscitation, and concern for hypoxic-ischemic brain injury. This case highlights the diagnostic value of real-time TEE in identifying structural causes of cardiac arrest. This case also illustrates the hemodynamic consequences of a rare and often fatal complication of aortic dissection.

## Introduction

Acute type A aortic dissection (ATAAD) carries an estimated mortality of 1-2% per hour in the first 24-48 hours without surgical intervention, making it one of the most time-critical cardiovascular emergencies encountered in clinical practice [1]. The spectrum of life-threatening complications includes cardiac tamponade, acute aortic insufficiency (AI), coronary ostial involvement with myocardial infarction, rupture, and malperfusion syndromes. However, mechanical obstruction of the left ventricular outflow tract (LVOT) by a proximal intimo-intimal intussuscepting dissection flap represents an exceptionally rare complication [2]. This phenomenon results in a unique and catastrophic hemodynamic profile that can be refractory to standard resuscitative measures and is rapidly fatal in the absence of emergent surgical intervention.

While transesophageal echocardiography (TEE) is not the primary diagnostic test for stable ATAAD patients, it is particularly useful in unstable/peri-arrest patients. TEE can localize the dissection flap, determine the severity and mechanism of AI, evaluate aortic valve function, and assess for LVOT obstruction (subvalvular and supravalvular), cardiac tamponade, and wall motion abnormalities [2].

We present this case to highlight a rare complication of ATAAD, describe its pathophysiology and hemodynamic consequences, and discuss the diagnostic and therapeutic implications for clinicians managing refractory cardiac arrest in known or suspected ATAAD.

## Case presentation

A 26-year-old woman with an unknown subtype of Ehlers-Danlos syndrome was transferred via air medical transport to our tertiary academic medical center after the acute onset of severe chest and back pain prior to presentation. Imaging from the outside facility had identified a large ATAAD with proximal aortic involvement, prompting emergent transfer for cardiothoracic surgical evaluation. En route, the patient required continuous bilevel positive airway pressure for acute hypoxemic respiratory failure and an esmolol infusion for impulse control. The flight crew reported a stable and uneventful transport.

Upon arrival at our helipad, the patient developed a witnessed pulseless electrical activity (PEA) cardiac arrest. The receiving emergency physicians initiated immediate advanced cardiovascular life support (ACLS). The patient was intubated and shortly thereafter developed copious frothy bloody secretions. TEE showed a well-filled left ventricle, no pericardial fluid, an anteriorly directed AI jet, and a large proximal aortic dissection. Throughout diastole, the redundant intimal flap was seen prolapsing across the aortic valve plane into the subvalvular LVOT, mechanically disrupting leaflet coaptation and producing AI; during systole, the flap was driven proximally into the aortic root, where it partially obstructed left ventricular outflow and impeded forward stroke volume (Figures 1-2).

**Figure 1:**
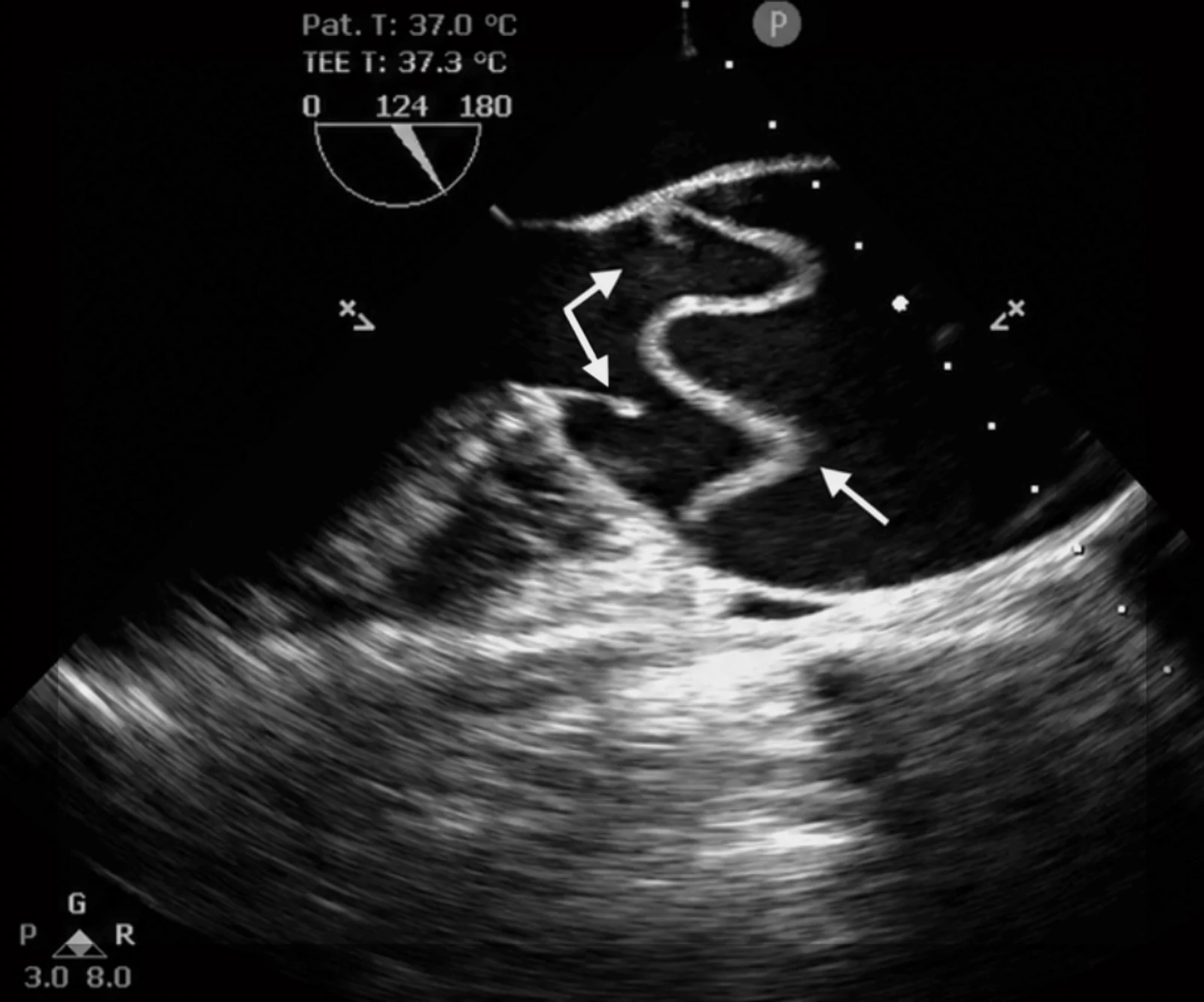
2D TEE mid-esophageal aortic valve long-axis view (124°) Mid-systolic frame during cardiopulmonary resuscitation demonstrating aortic valve (double arrow) opening with antegrade prolapse of the dissection flap (single arrow) into the aortic root. 2D: two-dimensional, TEE: transesophageal echocardiography

**Figure 2:**
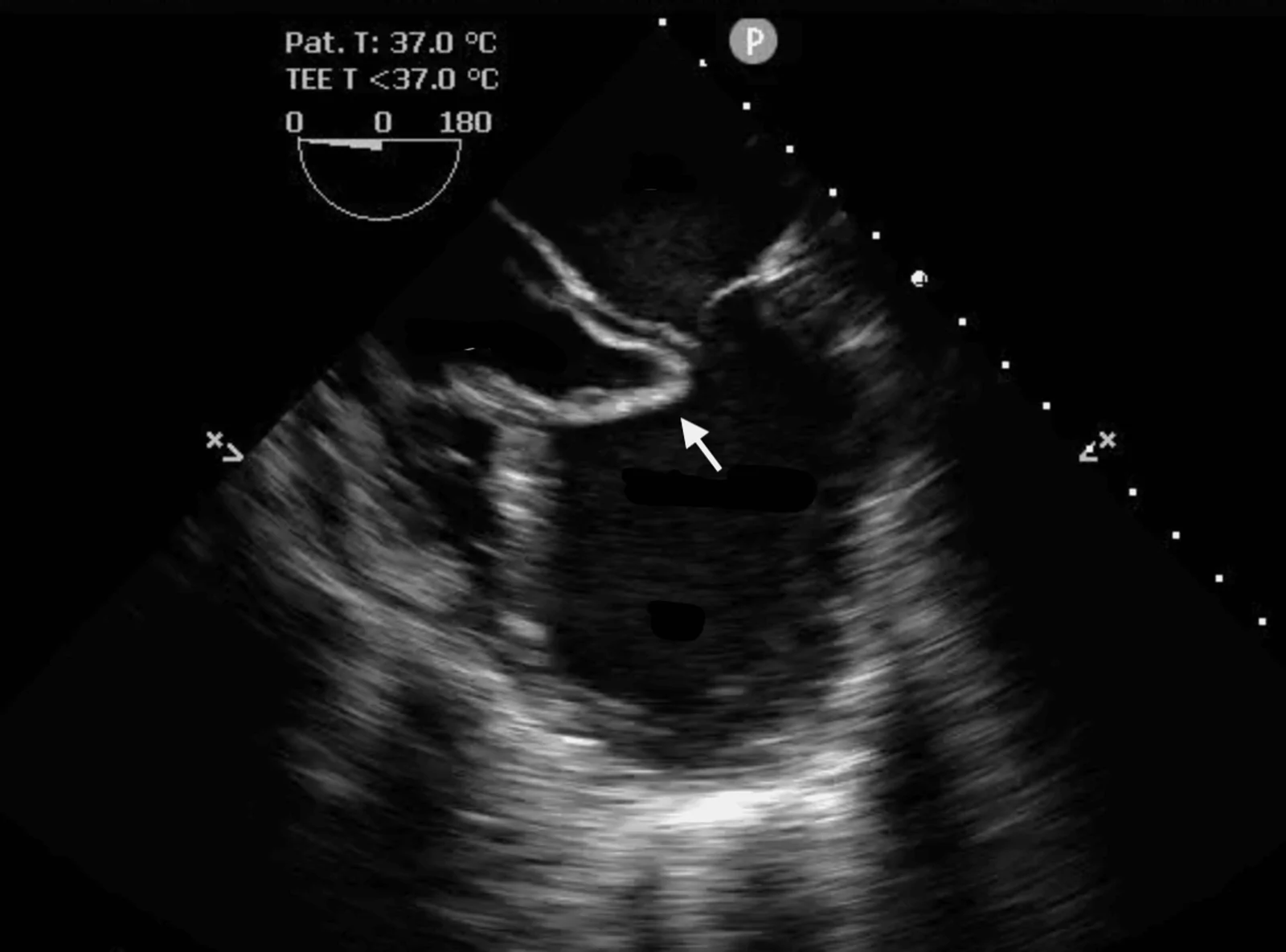
2D TEE mid-esophageal five-chamber view (0°) End-diastolic frame during cardiopulmonary resuscitation demonstrating retrograde prolapse of the dissection flap (arrow) into the LVOT. 2D: two-dimensional, LVOT: left ventricular outflow tract, TEE: transesophageal echocardiography

Over the subsequent 40 minutes, return of spontaneous circulation (ROSC) was achieved twice. Each time, there was a brief return of organized electrical activity and a weakly palpable pulse, followed quickly by recurrent hemodynamic collapse, PEA rhythm, and cardiac arrest. TEE performed during ROSC while on vasopressin and norepinephrine infusions demonstrated severe qualitative biventricular hypokinesis, spontaneous echo contrast in the left atrium and right ventricle, and a diminutive pulsed-wave Doppler spectral profile through the subvalvular LVOT (Figures 3-5). CPR was performed initially by providers, then transitioned to a mechanical CPR device. Epinephrine was given every three to five minutes, along with a single dose of sodium bicarbonate and calcium chloride. The recurrent, non-sustained ROSC despite resuscitative measures suggested an ongoing structural/hemodynamic cause that conventional ACLS alone could not correct.

**Figure 3:**
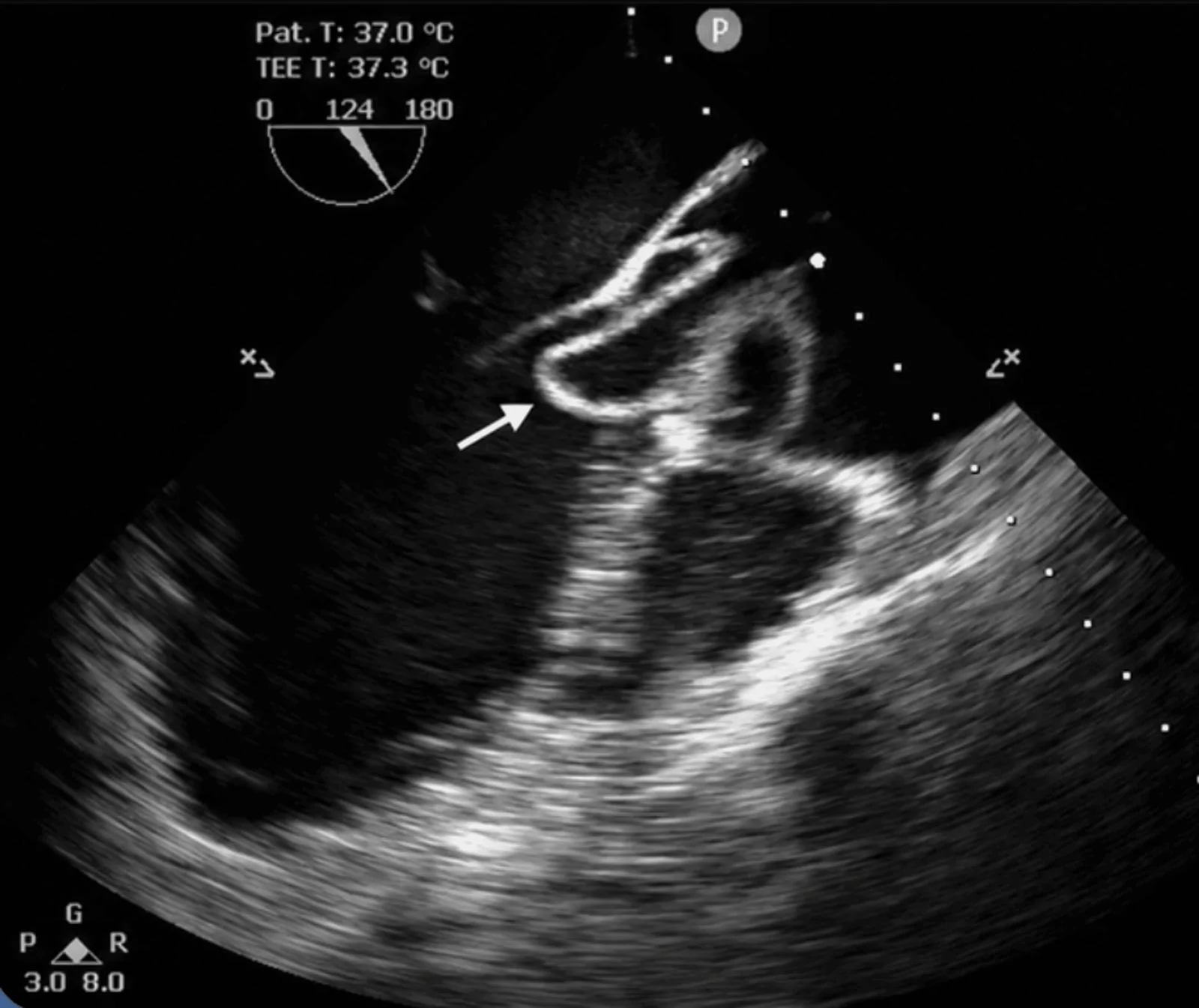
2D TEE mid-esophageal long-axis view (124°) Late diastolic frame during ROSC, showing retrograde prolapse of the dissection flap (arrow) into the LVOT. 2D: two-dimensional, LVOT: left ventricular outflow tract, TEE: transesophageal echocardiography, ROSC: return of spontaneous circulation

**Figure 4:**
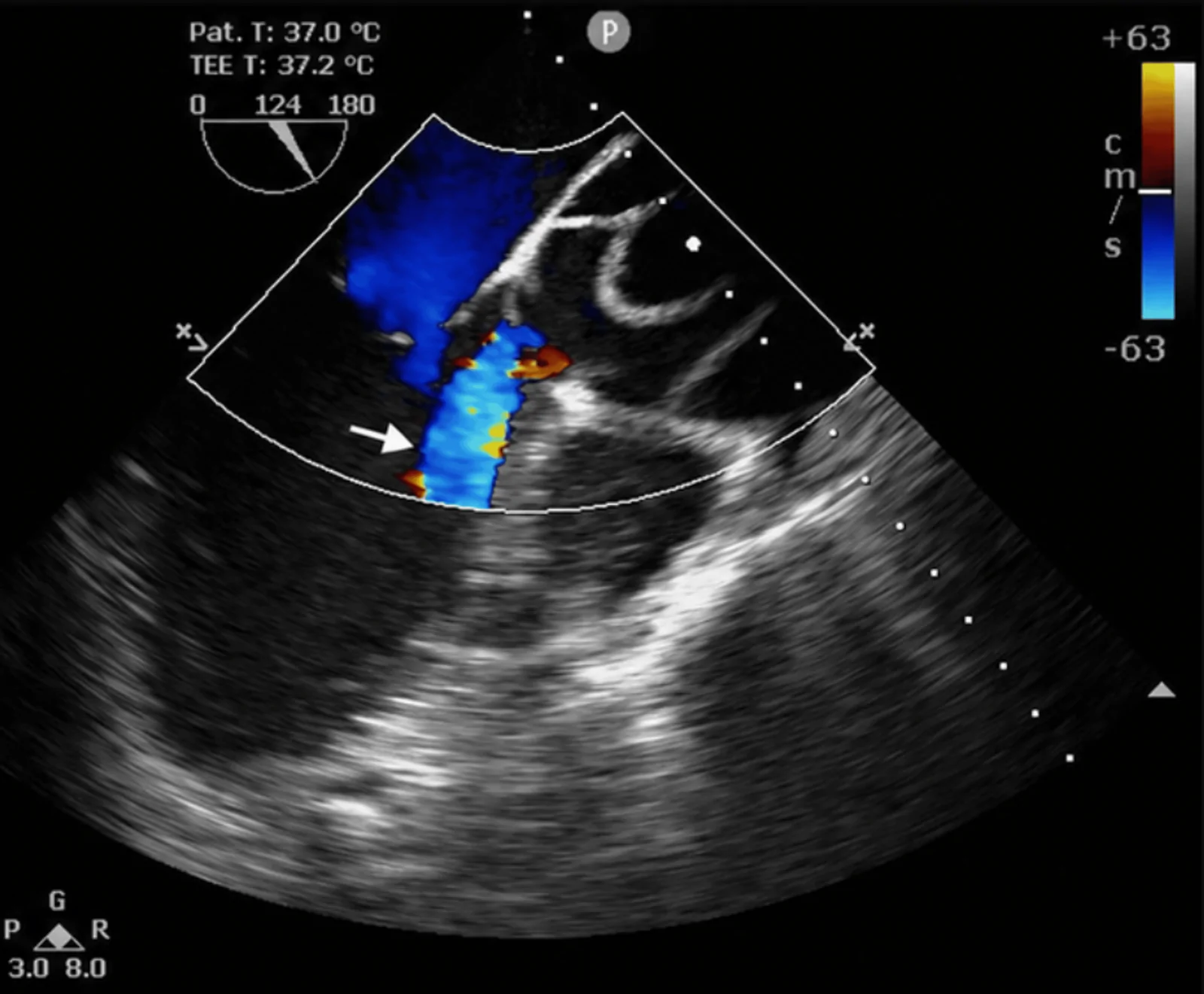
2D TEE mid-esophageal long-axis view (124°) Early diastolic frame during ROSC with color flow Doppler over the aortic and mitral valves, showing an AI jet (arrow). The size of the anteriorly directed AI jet likely underestimates the severity of regurgitation because of low cardiac output and near-equalization of pressure between the cardiac chambers during the peri-arrest period. 2D: two-dimensional, AI: aortic insufficiency, TEE: transesophageal echocardiography, ROSC: return of spontaneous circulation

**Figure 5:**
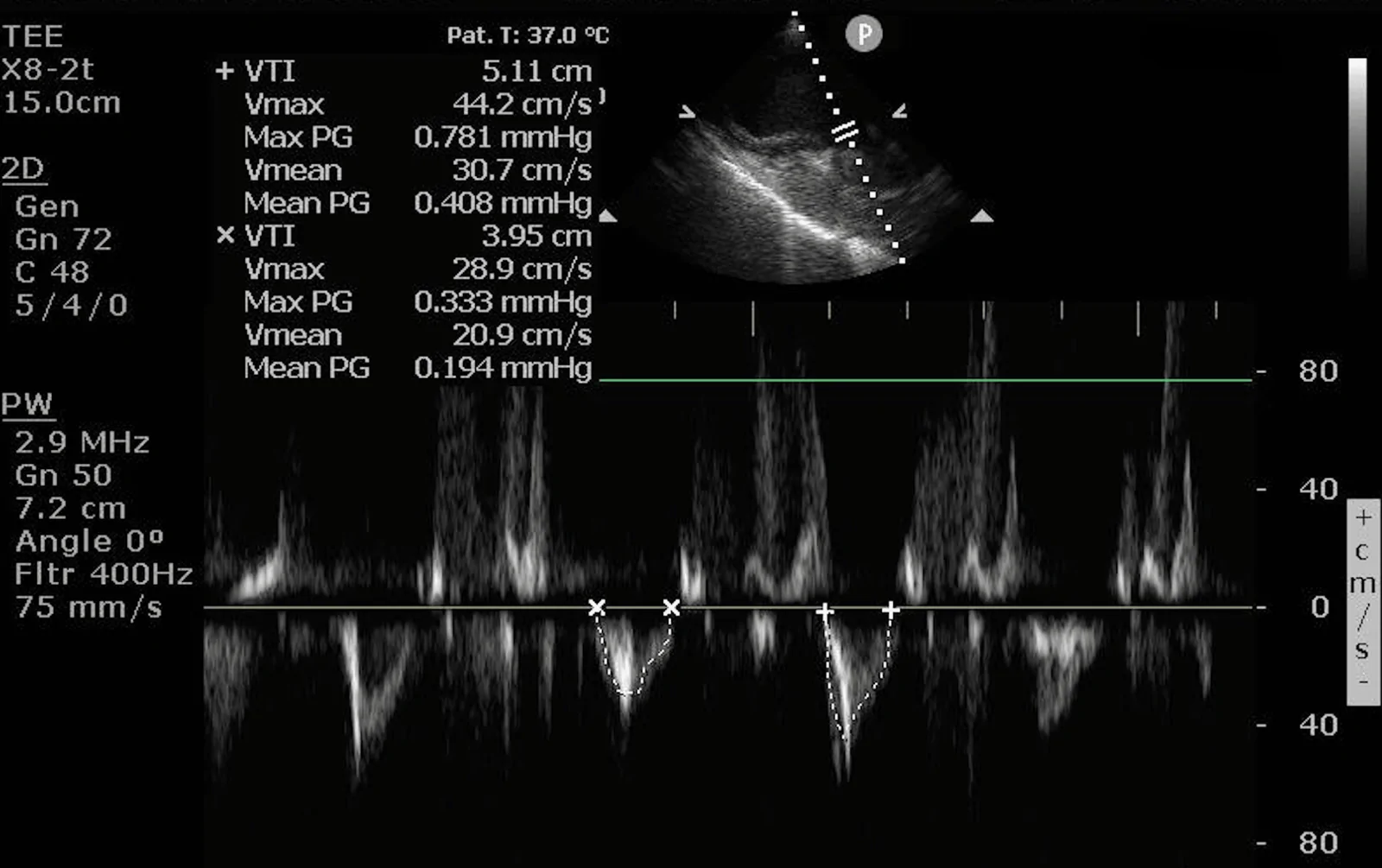
2D TEE transgastric long-axis view (120°) Pulsed-wave Doppler through the LVOT during ROSC shows a diminutive spectral Doppler profile, suggesting low stroke volume. 2D: two-dimensional, LVOT: left ventricular outflow tract, ROSC: return of spontaneous circulation, TEE: transesophageal echocardiography

After 45 minutes of resuscitation, sustained ROSC was not achieved. End-tidal carbon dioxide during the arrest had diminished from 15 mmHg to 8 mmHg. During brief periods of ROSC, there were no signs of life, including movement, pupillary reflexes, gag, or respiratory effort. PEA progressively degenerated into complete ventricular asystole. Considering the relative contraindication to veno-arterial extracorporeal membrane oxygenation (VA-ECMO) in aortic dissection, along with the prolonged low-flow state, recurrent arrest, persistently poor hemodynamics, and concern for severe hypoxic-ischemic brain injury, resuscitation efforts were terminated, and the patient was pronounced dead.

## Discussion

Our case highlights several features, including an uncommon presentation of a relatively common disease, specific pathophysiological challenges, and the role of TEE in diagnosing proximal intimo-intimal intussusception in ATAAD.

Prolapse of an aortic dissection flap into the LVOT is rare, occurring in approximately 2% of ATAAD [3]. The condition has been described using several terms, including “intimo-intimal intussusception," "circumferential dissection with intimal intussusception," and "dynamic LVOT obstruction from dissection flap," reflecting the absence of a universally adopted nomenclature. Its true incidence is likely underestimated, as many affected patients die before a diagnosis is established. Identifying this finding requires real-time echocardiographic expertise during active hemodynamic compromise.

The mechanism of LVOT obstruction by a prolapsing dissection flap can be explained by aortic root hemodynamics throughout the cardiac cycle. Under normal physiological conditions, the aortic valve opens during systole and closes during diastole, with the LVOT serving as the conduit for left ventricular ejection. With a large, mobile proximal dissection flap, the pressure differential between the false lumen and the left ventricular cavity during diastole can drive the flap across the aortic annulus plane and into the subvalvular space. This process disrupts aortic valve leaflet coaptation, resulting in AI, left ventricular volume and pressure overload, acute cardiogenic pulmonary edema, and impaired diastolic coronary perfusion [4]. During systole, the pressure differential across the aortic valve/LVOT results in antegrade prolapse of the dissection flap. In systole, the dissection flap acts as a supravalvular obstruction, compromising forward flow to the rest of the body, further exacerbated by acute left ventricular volume overload. The coexistence of impaired coronary perfusion, systemic malperfusion, and hypoxemia significantly limits the ability to achieve ROSC, maintain cerebral perfusion, or deliver effective cardiopulmonary resuscitation.

In addition to cardiac arrest, ATAAD can cause acute hemorrhagic pulmonary edema in the setting of acute AI. In this case, however, the severity of AI was likely underestimated because of low-flow states during CPR and after ROSC, with markedly elevated left atrial and left ventricular end-diastolic pressures and a reduced aorta-to-LV pressure gradient. Consequently, jet size may have substantially underestimated the degree of insufficiency. Despite this limitation, the pronounced diastolic coaptation defect caused by prolapse of the dissection flap suggested qualitatively severe AI. More broadly, quantitative and qualitative assessment of valvular insufficiency during profound low-flow states is inherently confounded and may not accurately reflect true regurgitant severity [5].

This case underscores the role of real-time TEE in managing cardiac arrest with suspected structural or mechanical etiology. Transthoracic echocardiography is frequently limited by body habitus, chest compressions, and probe positioning during active resuscitation. TEE, performed by a trained operator during ongoing ACLS, often provides superior, continuous cardiac imaging, does not interfere with compressions, and can simultaneously characterize aortic pathology, ventricular function, valvular integrity, and pericardial effusion [6-7]. The mid-esophageal long-axis view is particularly useful for visualizing the LVOT, aortic valve, and proximal ascending aorta. Intensivists, anesthesiologists, and emergency physicians with advanced TEE training are uniquely positioned to perform this diagnostic maneuver during resuscitation, as shown by the experience of several programs [8-10].

Given the mechanical and potentially reversible etiology of cardiac arrest in ATAAD with LVOT obstruction, extracorporeal cardiopulmonary resuscitation warrants consideration as a bridge to emergent surgical intervention in select cases. By bypassing the obstructed LVOT entirely and providing systemic flow via an extracorporeal circuit, VA-ECMO might restore cerebral and systemic perfusion. This could allow hemodynamic stabilization and bridge to emergent cardiopulmonary bypass and aortic repair.

However, significant cautions apply. Anticoagulation required for VA-ECMO initiation may exacerbate hemopericardium and precipitate tamponade in ATAAD. Further, VA-ECMO may worsen dissection-associated severe AI, further increasing left atrial pressure and potentially causing rapid pulmonary edema and hemorrhage. Additionally, femoral arterial cannulation carries a risk of false lumen access, potentially worsening malperfusion, and may be precluded if the dissection involves the iliofemoral vessels. The current Extracorporeal Life Support Organization guidelines consider greater-than-mild AI a contraindication due to the risk of severe left ventricular distension and pulmonary edema, and the American Heart Association explicitly lists aortic dissection as a contraindication [11-12]. Despite successful reports of using VA-ECMO for ATAAD, these risks must be weighed against the near-certain mortality of refractory cardiac arrest [13-14]. In our case, after multidisciplinary discussion among the treating team, cardiac surgeon, and ECMO team, and given concern for cerebral hypoxia due to prolonged cardiac arrest, our patient was deemed not to be a surgical candidate.

## Conclusions

Our case serves as a reminder of the catastrophic aortic complications that can occur in young patients with connective tissue disorders, including Marfan syndrome, Loeys-Dietz syndrome, and vascular Ehlers-Danlos syndrome. The hemodynamic profile of an intimo-intimal intussuscepting dissection flap can produce a hemodynamic state poorly responsive to conventional ACLS because the underlying structural obstruction and AI remain uncorrected. Real-time TEE was essential in establishing this mechanism during active cardiac arrest.
